# Marine Macroalga-Mediated Green Synthesis of Silver Nanoparticles Using *Ulva reticulata* for Sunlight-Assisted Photocatalytic Degradation of Synthetic Dyes

**DOI:** 10.3390/nano16191261

**Published:** 2026-10-07

**Authors:** Ganesan Ezhumalai, Harishna A. Ajay, Golap Chandra Singha, Sathiyaraj Srinivasan, Rajkumar Renganathan

**Affiliations:** 1 Department of Environmental Sciences, School of Life Sciences, Bharathiar University, Coimbatore 641046, Tamil Nadu, India; ezhumalai9695@gmail.com (G.E.); harishnaajay2000@gmail.com (H.A.A.); golapsingha6@gmail.com (G.C.S.); 2 Department of Bio & Environmental Technology, College of Natural Science, Seoul Women’s University, Seoul 01797, Republic of Korea

**Keywords:** *Ulva reticulata*, marine macroalga, silver nanoparticles, green synthesis, biogenic nanoparticles, photocatalytic degradation, synthetic dyes, wastewater treatment

## Abstract

Marine macroalgae provide renewable biological resources for the eco-friendly synthesis of functional nanomaterials for environmental remediation. This study synthesized silver nanoparticles (AgNPs) using an aqueous extract of the green macroalga *Ulva reticulata* and evaluated their sunlight-driven photocatalytic activity against synthetic dyes. Five seaweed species were screened for AgNP synthesis, and *U. reticulata* was selected based on visible color change and UV–visible spectral response. The effects of light conditions, incubation time, AgNO_3_-to-extract ratio, temperature, pH, and AgNO_3_ concentration were optimized. Maximum AgNP synthesis was achieved under light conditions using a 7:3 ratio of AgNO_3_ solution to algal extract, 1 mM AgNO_3_, pH 8, and incubation at 60 °C for 24 h. UV–visible spectroscopy showed a characteristic surface plasmon resonance peak at approximately 440 nm. FTIR analysis indicated that algal functional groups contributed to silver ion reduction and nanoparticle stabilization. SEM analysis showed predominantly spherical AgNPs with an average size of 3.1 nm, while EDS analysis confirmed the presence of elemental silver. The biosynthesized AgNPs degraded methylene blue, methyl orange, aniline blue, and rose bengal under sunlight, achieving 60–90% decolorization after 24 h. These findings support *U. reticulata* as a promising marine biological platform for sustainable AgNP synthesis and dye-contaminated wastewater treatment.

## 1. Introduction

Synthetic organic dyes are widely used in the textile, paper, plastic, pharmaceutical, food, and cosmetic industries. Among these, the textile sector is one of the major sources of dye-containing wastewater because large volumes of water and chemically stable dyes are used during dyeing and finishing processes [1,2,3]. Many synthetic dyes are resistant to light, heat, oxidation, and microbial degradation, allowing them to persist in aquatic environments after discharge. Their release into water bodies can reduce light penetration, inhibit photosynthesis, decrease dissolved oxygen levels, and affect aquatic organisms. In addition, several dye compounds and their breakdown products have been associated with toxic, mutagenic, or carcinogenic effects [3,4,5]. Therefore, the development of efficient and environmentally compatible approaches for dye removal remains an important research need.

Several physical, chemical, and biological methods have been used to remove dye pollutants from wastewater, including adsorption, coagulation, membrane filtration, phytoremediation, and microbial degradation. However, these approaches can be limited by high operational costs, incomplete degradation, slow reaction rates, generation of secondary sludge, or low efficiency against structurally complex dyes [6,7,8]. Photocatalytic degradation using nanomaterials has gained attention as an alternative strategy because it can promote the breakdown of dye molecules through light-induced reactive species, potentially converting complex organic pollutants into simpler and less harmful products [9,10].

Silver nanoparticles (AgNPs) are among the most widely studied metal nanoparticles because of their nanoscale size, high surface-area-to-volume ratio, surface plasmon resonance, catalytic activity, and antimicrobial properties [11,12]. These properties make AgNPs useful for environmental remediation, particularly in the treatment of dye-contaminated water. Conventional physical and chemical methods for AgNP synthesis often require high energy input, expensive equipment, or hazardous reducing and stabilizing agents. In contrast, green synthesis provides a safer and more sustainable route by using biological materials as reducing and capping agents, consistent with the principles of green chemistry [13,14]. Recent advances in biomass valorization have demonstrated that biological wastes, such as fruit residues, can serve as sustainable resources for the green synthesis of Ag and Au nanoparticles, thereby reducing the need for toxic substances and organic solvents. Biomass valorization through green and effective conversion approaches, such as the transformation of cellulose into valuable chemicals, has also been reported [15].

Marine macroalgae are promising biological resources for green nanoparticle synthesis because they are renewable, abundant, and rich in functional biomolecules. Seaweeds contain polysaccharides, proteins, amino acids, phenolic compounds, flavonoids, pigments, vitamins, and minerals, which can participate in metal ion reduction and nanoparticle stabilization [16,17]. The composition of these biomolecules varies among algal species; therefore, nanoparticle synthesis efficiency, stability, size, and functional activity are strongly influenced by the biological source and reaction conditions. Previous studies have reported the synthesis of metal nanoparticles using different seaweeds, including species of *Caulerpa*, *Padina*, *Sargassum*, and *Ulva*, with potential applications in photocatalysis, antimicrobial activity, and wastewater treatment [18,19,20,21,22].

Ag-based nanostructures can efficiently eliminate organic contaminants by visible light-induced degradation, although many such systems require intricately tailored composites [23]. Several macroalgae have been investigated for the synthesis of nanoparticles; however, *Ulva reticulata* optimized AgNP production and its use in the sunlight-driven degradation of multiple synthetic dyes are still not fully documented. In particular, systematic evaluation of synthesis parameters such as light conditions, incubation time, algal extract-to-silver nitrate ratio, temperature, pH, and AgNO_3_ concentration is important for improving reproducibility and nanoparticle performance. In this study, five seaweed species were screened for AgNP synthesis, and *U. reticulata* was selected for further optimization. The synthesized AgNPs were characterized using UV–visible spectroscopy, FTIR, SEM, EDS, and zeta potential analyses. Their photocatalytic activity was then evaluated against methylene blue, methyl orange, aniline blue, and rose bengal under sunlight irradiation. Furthermore, *U. reticulata* is abundant in marine environments and can be obtained at relatively low cost. This study highlights the potential of *U. reticulata* as a marine biological platform for sustainable AgNP synthesis and dye-contaminated wastewater remediation. The present investigation demonstrates a sustainable approach for the green synthesis of silver nanoparticles using *U. reticulata*, avoiding hazardous chemicals and using algal material as both a reducing and stabilizing agent. In addition, the synthesized silver nanoparticles were evaluated for photocatalytic applications, demonstrating their potential for wastewater remediation. The approach may also support future scale-up by linking green synthesis with functional application.

## 2. Materials and Methods

### 2.1. Sample Collection

Fresh seaweed samples were collected from the Mandapam coastal region, Ramanathapuram district, Tamil Nadu, India. The collected samples were washed thoroughly with seawater at the collection site to remove sand, sediment, epiphytes, and other surface-associated debris and then transported to the laboratory in sterile containers. In the laboratory, the samples were rinsed several times with distilled water to remove remaining impurities. The seaweed samples were identified based on morphological characteristics using standard taxonomic references [24,25,26]. Representative specimens were photographed and retained in the laboratory for reference. After identification, the samples were shade-dried at room temperature for 2–4 days, ground into a fine powder, and stored in airtight containers for further experimental use.

### 2.2. Preparation of Aqueous Seaweed Extract

The aqueous seaweed extract was prepared following previously described hot-water extraction methods for seaweed-mediated nanoparticle synthesis, with slight modifications [27]. Briefly, 5 g of dried seaweed powder was mixed with 100 mL of deionized water and heated at 60 °C for 15 min. The mixture was cooled to room temperature and filtered to remove solid residues. The resulting aqueous extract was stored at −15 °C and used for the biosynthesis of silver nanoparticles.

### 2.3. Optimization of U. reticulata-Mediated Silver Nanoparticle Synthesis

Five seaweed species, namely *Ulva reticulata*, *Ulva lactuca*, *Codium tomentosum*, *Sargassum swartzii*, and *Halymenia floresia*, were initially screened for AgNP synthesis using the same extract preparation and reaction conditions. Among them, *U. reticulata* was selected for further optimization based on visible color change and UV–visible spectral absorbance. The synthesis of silver nanoparticles was optimized using a one-factor-at-a-time approach, as described previously [28], with slight modifications. Reaction parameters were evaluated based on visual color change and UV–visible absorbance to determine their influence on AgNP formation. Initially, 8 mL of 1 mM AgNO_3_ solution was mixed with 2 mL of *U. reticulata* aqueous extract and incubated under light and dark conditions. Using the same reaction ratio, the effect of incubation time was examined at 0, 3, 6, 9, 12, and 24 h. To determine the optimal precursor-to-extract ratio, AgNO_3_ solution and algal extract were mixed at different ratios of 9:1, 8:2, 7:3, 6:4, 5:5, 4:6, 3:7, 2:8, and 1:9. The effect of temperature was tested at 16, 37, and 60 °C. The influence of pH was evaluated over a range of pH 7–12, with the pH adjusted using 1 N NaOH or 1 N HCl. Finally, the effect of AgNO_3_ concentration was assessed using 0.1, 1, 2, 3, and 4 mM AgNO_3_ solutions mixed with algal extract. After incubation, AgNP formation was monitored by UV–visible spectroscopy (LMSP-UV1900, LABMAN, Chennai, India) over the wavelength range of 300–700 nm.

### 2.4. Characterization of Synthesized Silver Nanoparticles

The formation of AgNPs was monitored using UV–visible spectroscopy over a wavelength range of 300–700 nm at different incubation intervals. For further characterization, the synthesized AgNP suspension was centrifuged at 20,000 rpm for 15 min, and the resulting pellet was washed three times with deionized water to remove unbound biomolecules and residual ions. The collected AgNP pellet was dried at room temperature and used for FTIR, SEM, EDS, and zeta potential analyses. For FTIR (ATR/FTIR, JASCO4700, Hachioji, Japan) analysis, the dried AgNP powder was mixed with potassium bromide (KBr), pressed into pellets, and analyzed to identify the functional groups associated with nanoparticle reduction and stabilization. For SEM (FESEM QUANTA FEG250/BRUKER EDS QUANTAX, Berlin, Germany) analysis, the dried AgNP powder was mounted on a copper grid, coated with gold, and examined to determine surface morphology and particle structure. EDS (BRUKER EDS QUANTAX, Berlin, Germany) analysis was performed to confirm the elemental composition of the synthesized nanoparticles. Zeta potential analysis was used to evaluate the surface charge and colloidal stability of the AgNPs.

### 2.5. Photocatalytic Degradation of Dyes

The photocatalytic activity of the green-synthesized AgNPs was evaluated using four synthetic dyes: methylene blue, methyl orange, aniline blue, and rose Bengal. Approximately 8 mL of dye solution (100 mg/L) was placed in a 50 mL conical flask. The reaction was conducted at the natural pH of the dye solution, and 2 mL of the AgNP suspension was added to the dye solution. Before irradiation, the mixture was kept in the dark for 10 min to establish adsorption–desorption equilibrium. The reaction mixture was directly exposed to sunlight between 08:00 and 12:00 h. The experiments were conducted at an ambient temperature of 36 ± 2 °C. Samples were collected at different time intervals. The collected samples were centrifuged at 5000 rpm before analysis. Following that, the residual dye concentration was monitored by measuring absorbance using a UV–visible spectrophotometer. Control trials were maintained under dark conditions using the same dye concentration, catalyst dosage, reaction volume, and pH. A photolysis control containing dye solution without AgNPs was also exposed to sunlight under identical conditions.

The percentage of dye decolorization was calculated using the following equation:

% Decolorization = [(C_0_ − C)/C_0_] × 100

where C_0_ is the initial absorbance of the dye solution, and C is the absorbance after photocatalytic treatment.

### 2.6. Time-Dependent Decolorization of Synthetic Dyes

The time-dependent photocatalytic decolorization of methyl orange, methylene blue, aniline blue, and rose bengal was evaluated under natural sunlight irradiation. Briefly, 100 mL of each dye solution was prepared at a final concentration of 100 ppm and treated with the synthesized AgNPs. Dye solutions without AgNPs were maintained as controls. Both treated and control solutions were exposed to natural sunlight under identical conditions for a total of 8 h. At 1 h intervals, 2 mL aliquots were collected from each reaction mixture and centrifuged at 5000 rpm for 10 min to remove suspended nanoparticles. The absorbance of the collected supernatant was then measured using a UV–visible spectrophotometer (LMSP-UV.1900, Labman, Chennai, India) over a wavelength range of 300–800 nm to monitor dye decolorization over time. All experiments were performed in triplicate, and the results are presented as mean ± standard deviation.

## 3. Results

### 3.1. Screening of Seaweed Extracts for Silver Nanoparticle Synthesis

Five seaweed species, namely *Ulva reticulata*, *Ulva lactuca*, *Codium tomentosum*, *Sargassum swartzii*, and *Halymenia floresia*, were initially screened for their ability to mediate silver nanoparticle synthesis. The reaction mixtures were observed under light and dark incubation conditions, and nanoparticle formation was preliminarily assessed based on visible color change. Among the tested seaweed extracts, *U. reticulata* and *S. swartzii* showed a clear color change from pale yellow to brown under light conditions, indicating the reduction of silver ions and possible AgNP formation. No visible color change was observed for *U. lactuca*, *C. tomentosum*, or *H. floresia* under the tested conditions. In addition, none of the seaweed extracts showed a positive reaction under dark incubation. Based on the intensity of color development and UV–visible spectral response, *U. reticulata* was selected for further optimization of AgNP synthesis (Table 1).

### 3.2. Optimization of U. reticulata-Mediated AgNP Synthesis

**Figure 1:**
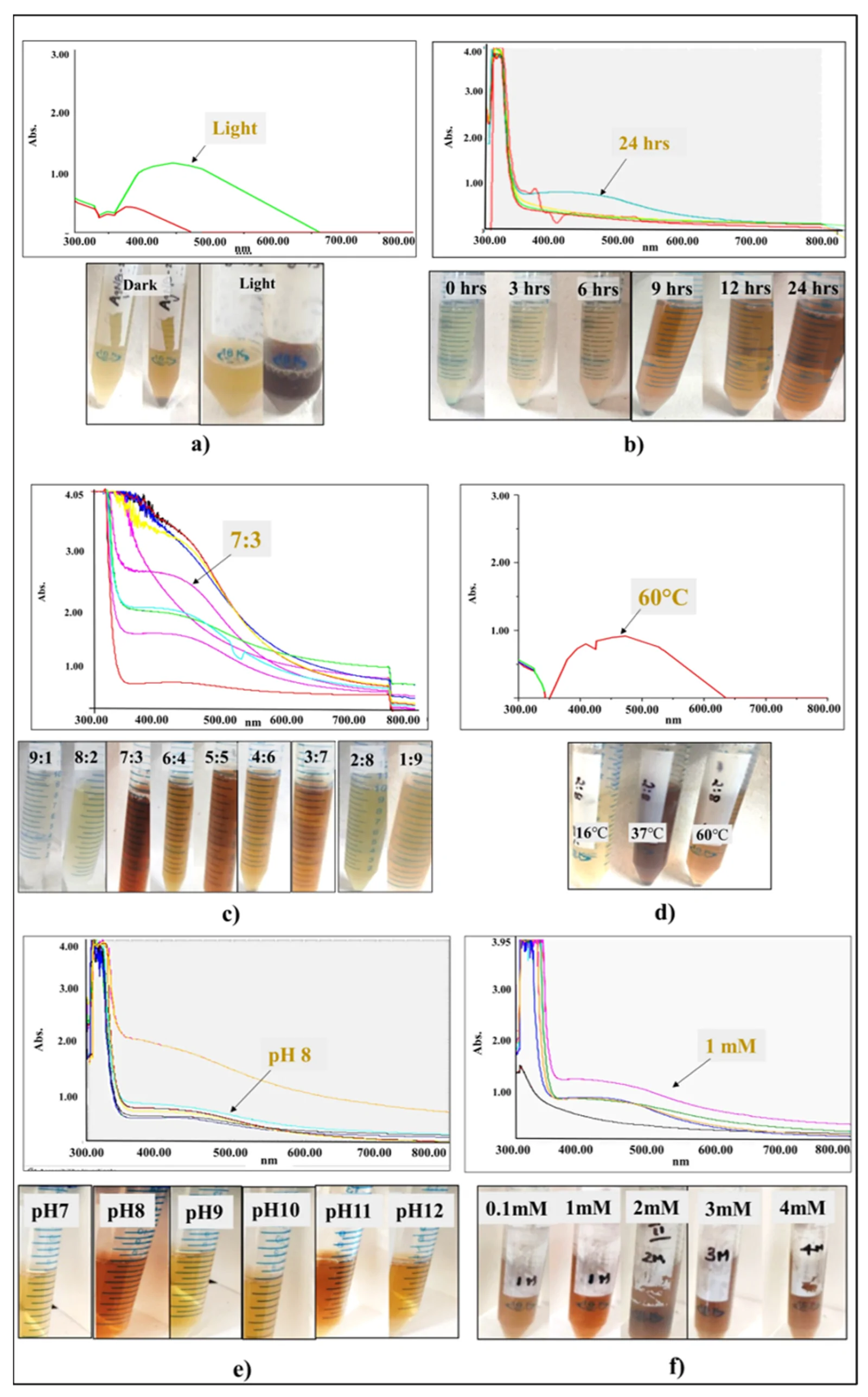
Optimization of AgNP synthesis using *Ulva reticulata* extract. Effects of (**a**) incubation condition (light and dark), (**b**) reaction time, (**c**) AgNO_3_-to-extract ratio, (**d**) temperature, (**e**) pH, and (**f**) AgNO_3_ concentration on nanoparticle formation monitored by UV–visible spectroscopy.

The synthesis of AgNPs using *U. reticulata* extract was optimized by evaluating different reaction parameters, including incubation conditions, reaction time, AgNO_3_-to-extract ratio, temperature, pH, and AgNO_3_ concentration. AgNP formation was monitored by visual color change and UV–visible absorbance. Among the incubation conditions, light exposure showed a stronger response than dark incubation, indicating more effective AgNP formation under light conditions. The absorbance intensity increased gradually with incubation time, with the highest response observed after 24 h. The AgNO_3_-to-extract ratio also influenced nanoparticle formation, and the 7:3 ratio produced the most distinct absorbance peak. Among the tested temperatures, 60 °C showed the strongest spectral response. The reaction was also affected by pH, with maximum AgNP formation observed at pH 8. Among the different AgNO_3_ concentrations, 1 mM produced the most suitable absorbance pattern for AgNP synthesis. Overall, the optimized conditions for *U. reticulata*-mediated AgNP synthesis were light incubation, a 24 h reaction time, a 7:3 AgNO_3_-to-extract ratio, 60 °C, pH 8, and 1 mM AgNO_3_. Under these conditions, the UV–visible spectrum showed a characteristic surface plasmon resonance peak at approximately 440 nm, confirming AgNP formation (Figure 1).

### 3.3. Biosynthesis of AgNPs Under Optimized Conditions

**Figure 2:**
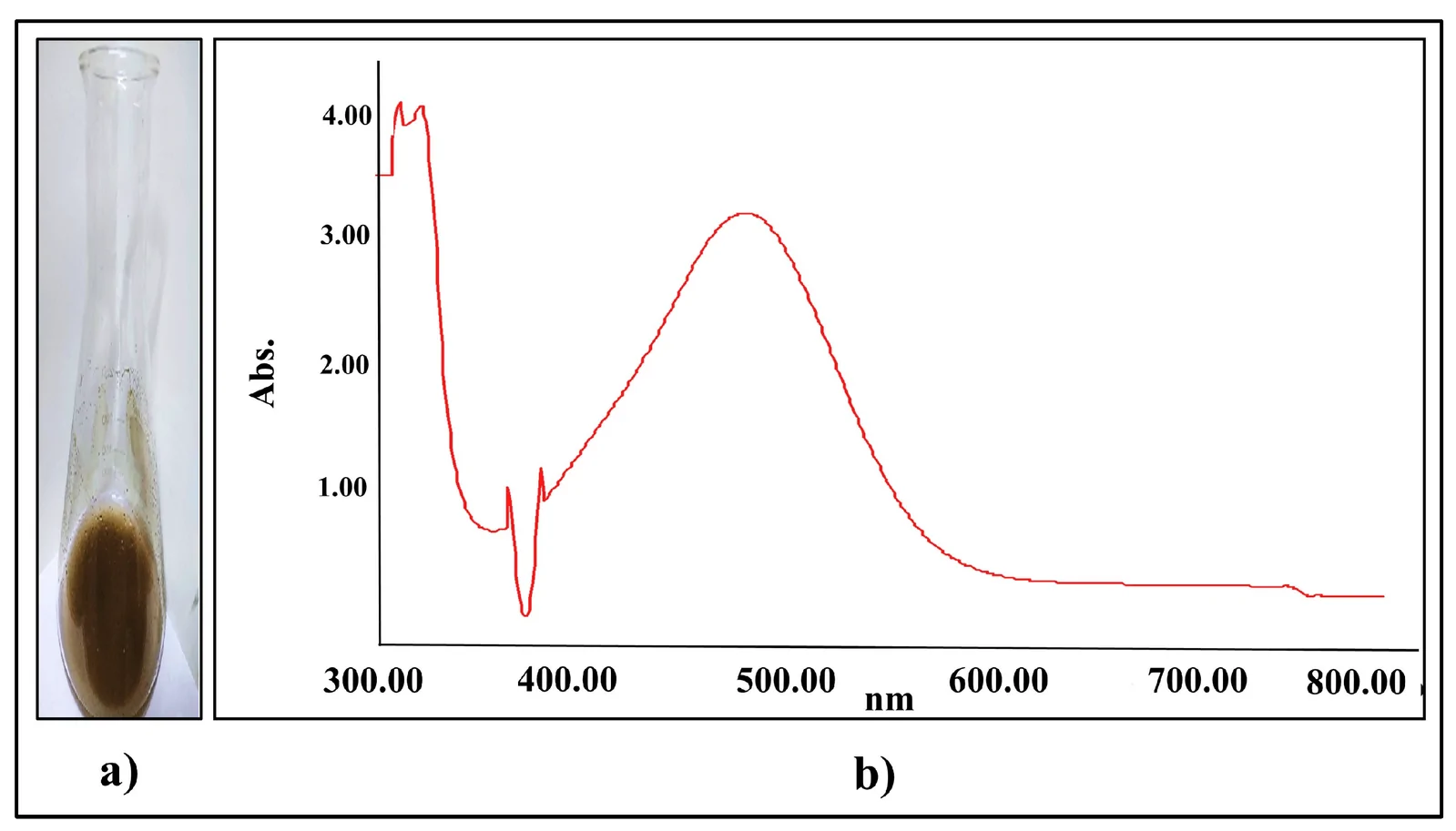
Biosynthesis of AgNPs using *Ulva reticulata* under optimized conditions: (**a**) Color change of the reaction mixture from pale yellow to brown following AgNP formation. (**b**) UV–visible spectrum showing the characteristic surface plasmon resonance peak at approximately 440 nm.

Based on the optimization results, AgNPs were synthesized using *U. reticulata* extract under the selected conditions: 1 mM AgNO_3_, a 7:3 AgNO_3_-to-extract ratio, pH 8, incubation at 60 °C for 24 h, and light exposure. During incubation, the reaction mixture changed from pale yellow to brown, indicating the reduction of Ag^+^ ions and the formation of AgNPs. In contrast, the control solutions, including AgNO_3_ solution without algal extract and algal extract without AgNO_3_, did not show comparable brown color development under the same incubation conditions. UV–visible spectroscopic analysis showed a characteristic surface plasmon resonance peak at approximately 440 nm, further supporting AgNP formation. Together, the visible color change, control comparison, and UV–visible spectral response verified the successful biosynthesis of AgNPs under the optimized reaction conditions (Figure 2).

### 3.4. FTIR Analysis of U. reticulata-Mediated AgNPs

**Figure 3:**
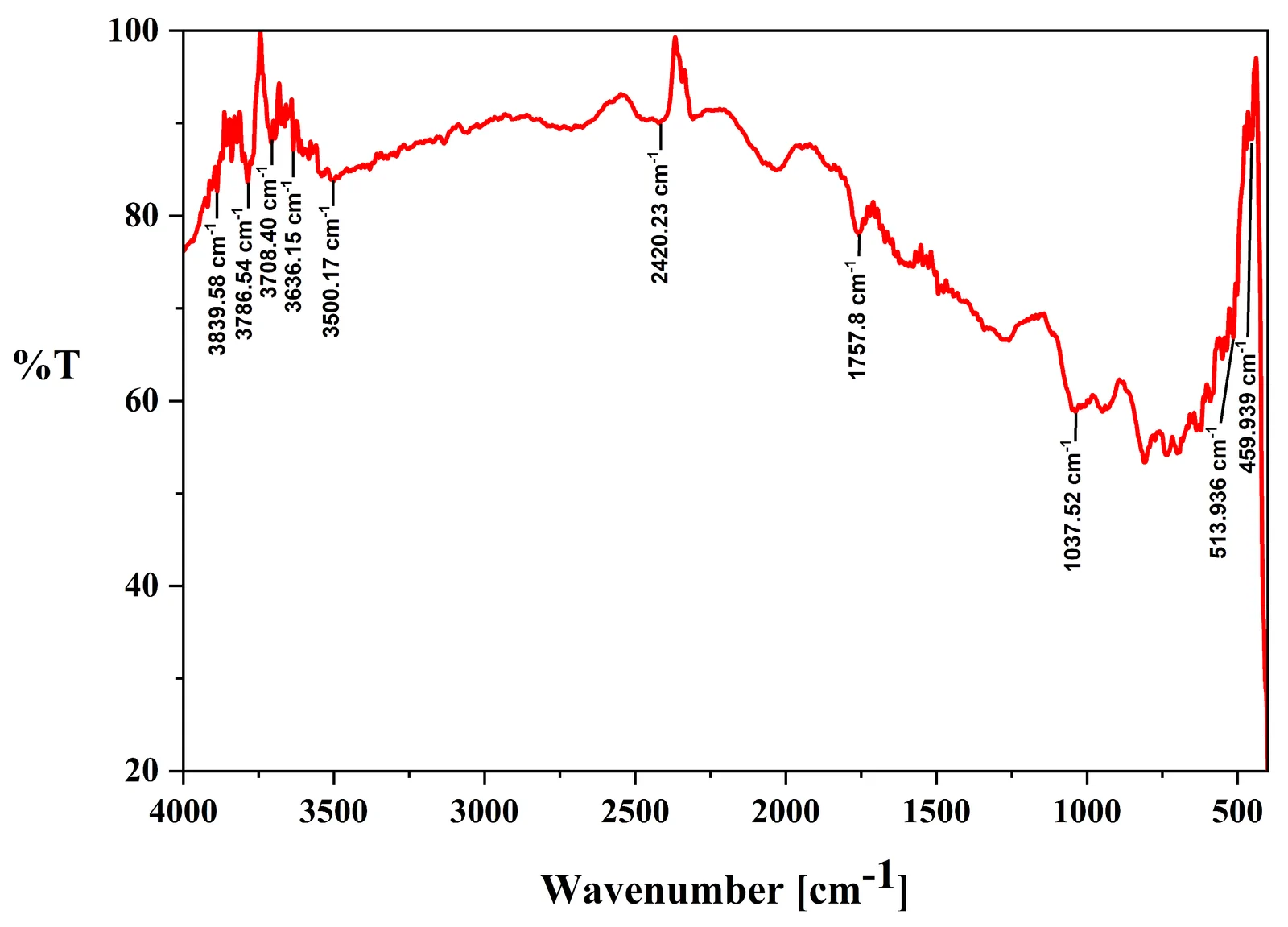
FTIR spectrum of biosynthesized AgNPs produced using *Ulva reticulata* extract, showing the major functional groups involved in nanoparticle reduction and stabilization.

FTIR analysis was performed to identify the functional groups associated with the biosynthesized AgNPs. The FTIR spectrum showed major peaks at 3839.58, 3786.54, 3708.40, 3635.16, 3500.17, 2420.23, 1758.80, 1037.52, 809.96, and 513.93 cm^−1^ (Figure 3). The broad absorption region between 3500 and 3700 cm^−1^ was assigned to O–H and N–H stretching vibrations, indicating the possible involvement of hydroxyl- and amine-containing biomolecules. The peak at 1758.80 cm^−1^ corresponded to C=O stretching vibrations, while the peak at 1037.52 cm^−1^ was associated with CO stretching, likely related to polysaccharides or other oxygen-containing algal metabolites. Peaks at 809.96 and 513.93 cm^−1^ may be attributed to bending or vibration regions associated with organic residues and metal-associated bonding. The previous study showed similar characteristics of the natural compound (1E, 6E)-1,7-bis(4-hydroxy-3-methoxyphenyl)-1,6-heptadiene-3,5-diene in the range of 1030 to 1627 cm^−1^ [29]. Therefore, these results suggest that functional groups present in the *U. reticulata* extract contributed to the reduction and stabilization of AgNPs.

### 3.5. Zeta Potential, Morphology, and Elemental Composition of AgNPs

**Figure 4:**
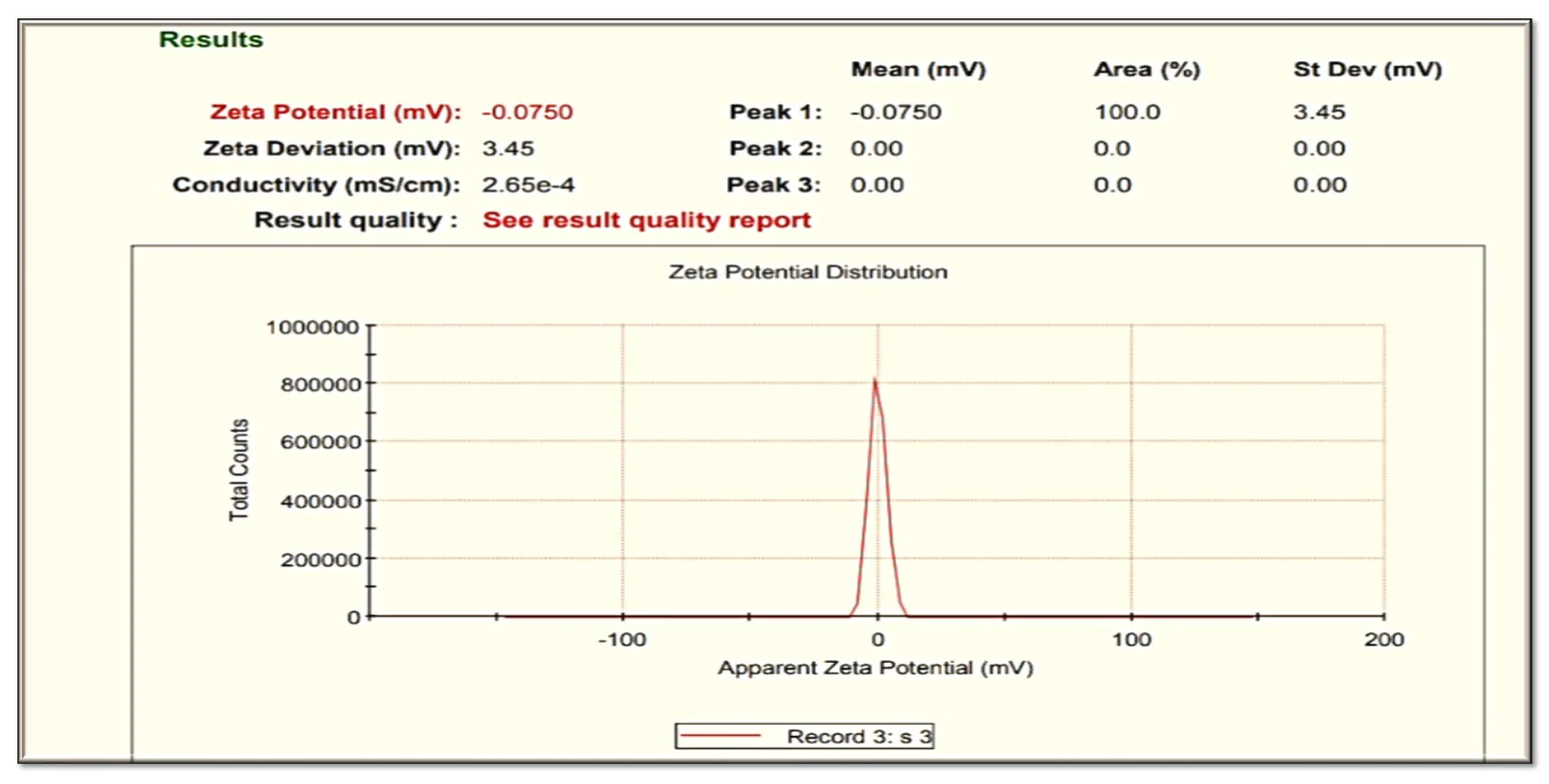
Zeta potential distribution of biosynthesized AgNPs synthesized using *Ulva reticulata* extract.

**Figure 5:**
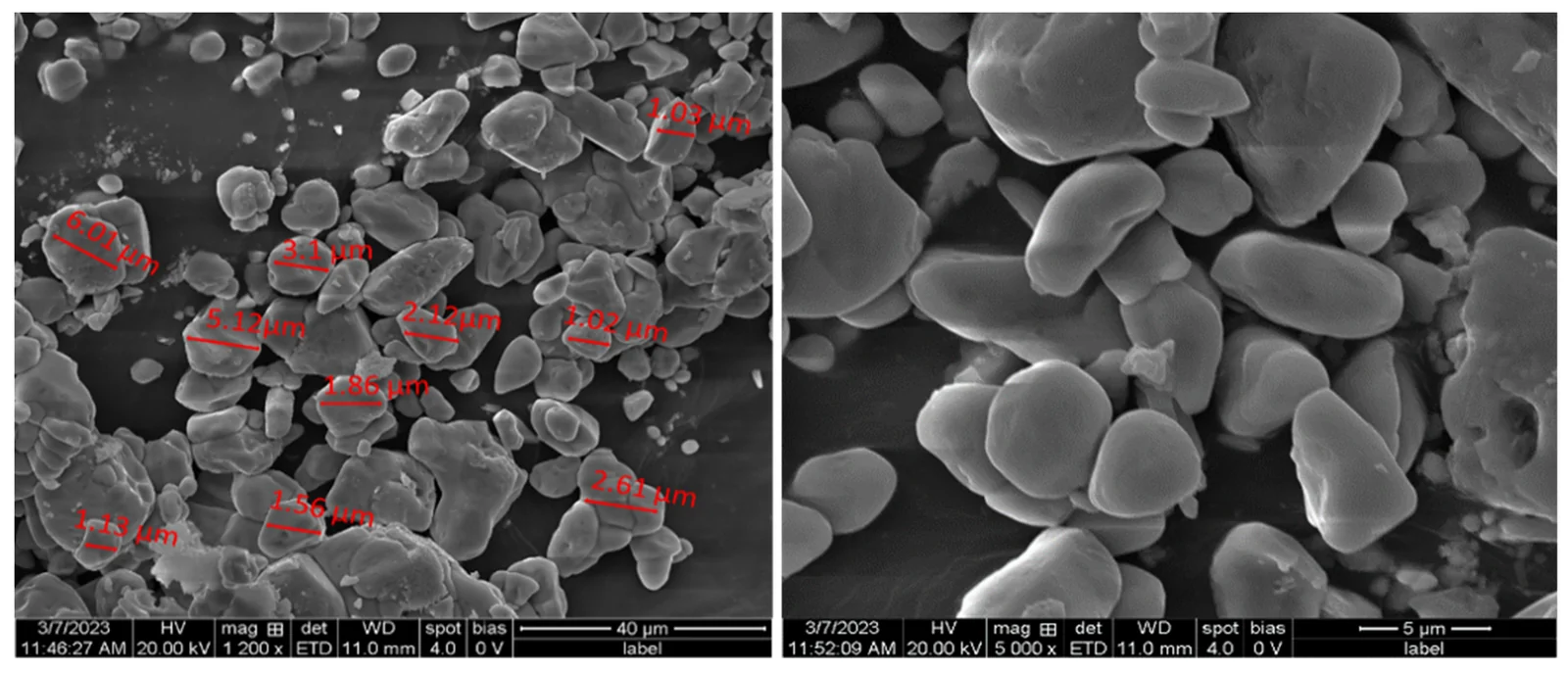
Scanning electron micrographs of biosynthesized AgNPs produced using *Ulva reticulata* extract showing predominantly spherical nanoparticles with an average particle size of approximately 3.1 nm.

**Figure 6:**
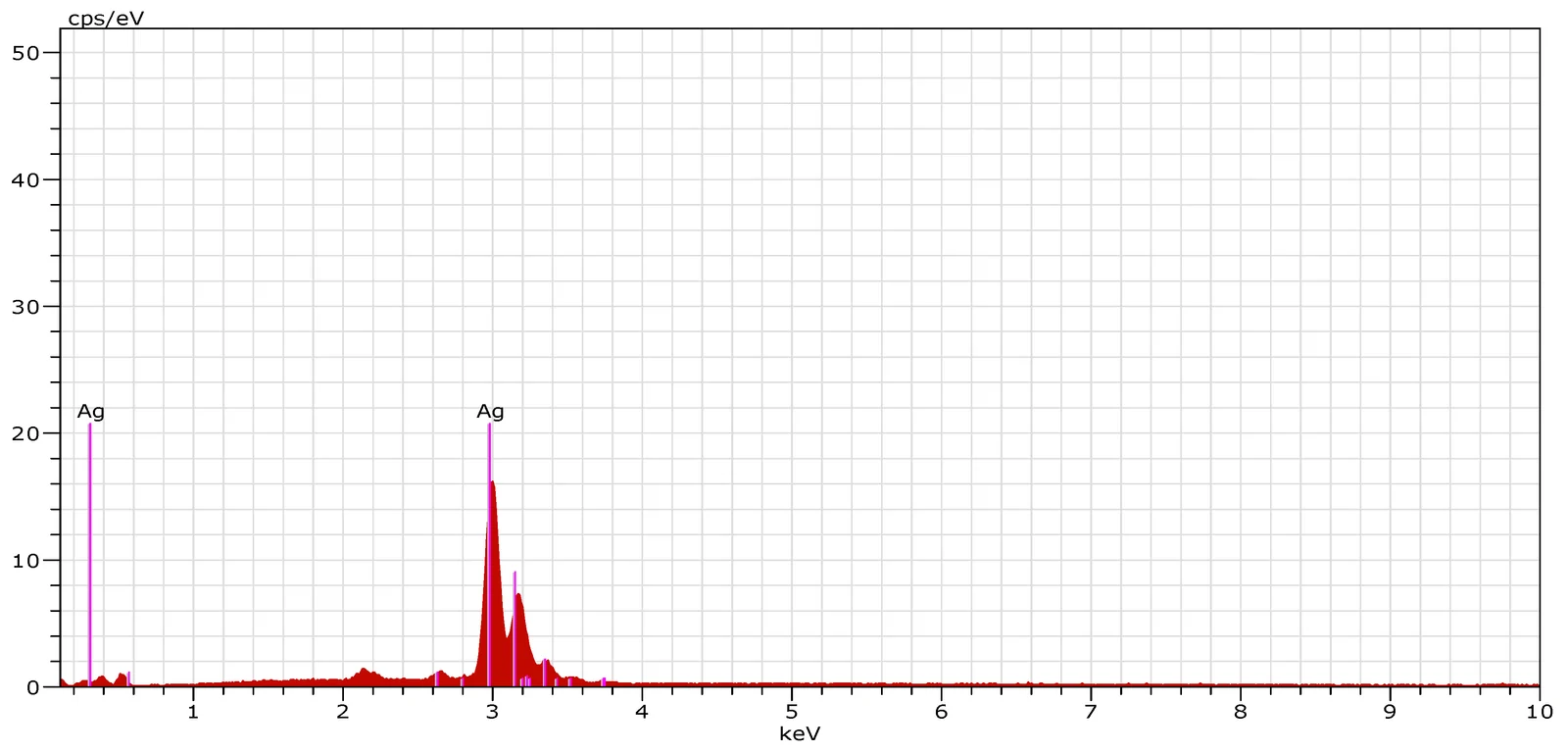
Energy-dispersive X-ray spectroscopy (EDS) spectrum of biosynthesized AgNPs confirming the elemental silver composition of the nanoparticles.

The surface charge, morphology, size, and elemental composition of the biosynthesized AgNPs were evaluated using zeta potential analysis, SEM, and EDS. Zeta potential analysis showed a value of −0.0750 ± 3.45 mV, indicating the surface charge characteristics of the synthesized AgNPs (Figure 4). Zeta potential analysis showed a value of −0.0750 ± 3.45 mV, indicating a near-neutral surface charge and weak electrostatic repulsion between the synthesized AgNPs (Figure 4). This indicates weak electrostatic stabilization and a potential tendency toward aggregation; however, biomolecules attached to the biogenic AgNP surface may provide additional steric stabilization. However, the biomolecules attached to the biogenic AgNP surface may provide greater steric stability. Therefore, the zeta-potential result should be interpreted in conjunction with the observed morphology and particle-size parameters rather than as a definitive indication of colloidal stability. The near-neutral zeta potential indicates weaker electrostatic stabilization than that reported for AgNPs with more negative zeta potentials. SEM analysis revealed predominantly spherical AgNPs, and particle size was estimated from SEM images by measuring approximately 100 particles. The synthesized AgNPs showed an average particle size of approximately 3.1 nm (Figure 5). The biosynthesized AgNPs showed irregular to spherical shapes with discernible particle aggregation, according to SEM examination. SEM results were primarily interpreted in terms of shape and aggregation since the observed micrometer-scale structures represent aggregated assemblies rather than individual nanoparticles. Drying during sample preparation and interactions with biomolecules associated with the biogenic synthesis may have contributed to such agglomeration. EDS analysis showed characteristic silver signals, with major peaks observed at approximately 0.3 and 3.0 keV, confirming the presence of elemental silver in the synthesized nanoparticles. Silver accounted for 74.70 wt% of the detected elements, with an atomic percentage of 100%, indicating that silver was the predominant elemental constituent of the nanoparticles (Figure 6). The presence of silver (Ag) in the produced nanoparticles was verified by EDX analysis, as evidenced by the distinctive Ag signal. The effective production of Ag-containing nanoparticles is supported by the conspicuous Ag peak. Although EDX cannot detect nanoparticle size or morphology on its own, the SEM morphology and the EDX results offer complementary evidence for AgNP production. For green-synthesized AgNPs made with biological extracts, comparable Ag signals have been documented. Together, the SEM and EDS results support the successful formation of predominantly spherical AgNPs using the *U. reticulata* extract.

### 3.6. Photocatalytic Decolorization of Synthetic Dyes

**Figure 7:**
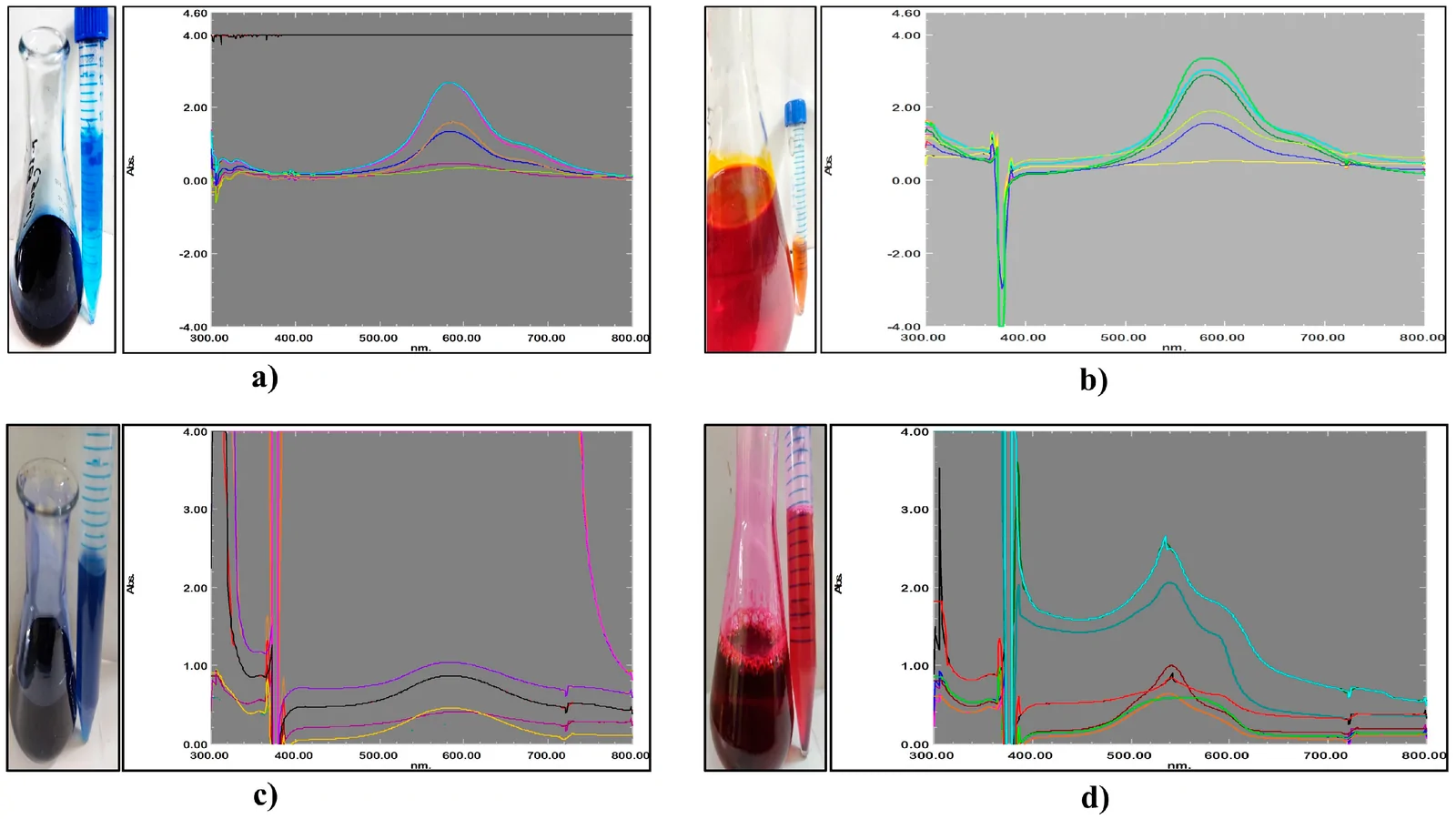
Time-dependent photocatalytic decolorization of synthetic dyes by biosynthesized AgNPs under natural sunlight irradiation. UV–visible absorption spectra recorded between 300 and 800 nm for (**a**) methylene blue, (**b**) methyl orange, (**c**) aniline blue, and (**d**) rose bengal.

The photocatalytic activity of the biosynthesized AgNPs was evaluated against four synthetic dyes: methylene blue, methyl orange, aniline blue, and rose bengal. Dye solutions treated with AgNPs were exposed to natural sunlight, and time-dependent changes in absorbance were monitored by scanning the UV–visible spectra over a wavelength range of 300–800 nm. A gradual decrease in absorbance was observed with increasing exposure time, indicating progressive dye decolorization in the presence of AgNPs. In contrast, the control dye solutions without AgNPs showed comparatively limited color reduction under the same sunlight conditions. After 24 h of sunlight exposure, the decolorization efficiencies of the AgNP-treated dye solutions were 90% for methylene blue, 85% for methyl orange, 70% for aniline blue, and 63% for rose bengal. These results indicate that *U. reticulata*-mediated AgNPs can promote sunlight-assisted decolorization of structurally different synthetic dyes (Figure 7).

## 4. Discussion

Marine macroalgae are promising biological resources for green nanoparticle synthesis because they are renewable, widely available, and rich in functional biomolecules. In the present study, five seaweed species were screened for AgNP synthesis, and *U. reticulata* was selected for further optimization based on visible color development and UV–visible spectral response. The difference in AgNP-forming ability among the tested seaweeds suggests that nanoparticle synthesis is strongly influenced by the biochemical composition of each algal extract. Seaweeds contain polysaccharides, proteins, amino acids, phenolic compounds, flavonoids, pigments, vitamins, and minerals, which can participate in the reduction of Ag+ ions and stabilization of newly formed nanoparticles [16,17,30]. Therefore, *U. reticulata* extract provides a simple aqueous system for AgNP production while reducing the need for harsh chemical reducing agents or synthetic stabilizers.

The optimization results showed that AgNP formation was affected by light conditions, incubation time, AgNO_3_-to-extract ratio, temperature, pH, and AgNO_3_ concentration. These factors influence biogenic nanoparticle synthesis by affecting the balance among silver ion availability, reducing biomolecules, and capping agents. In this study, maximum AgNP formation was observed under light exposure using a 7:3 AgNO_3_-to-extract ratio, 1 mM AgNO_3_, pH 8, and incubation at 60 °C for 24 h. The gradual increase in absorbance over time indicates progressive reduction of Ag^+^ ions and nanoparticle formation. The optimized AgNO_3_-to-extract ratio suggests that an appropriate balance between precursor and algal biomolecules is necessary for efficient synthesis. Similar effects of pH, temperature, metal salt concentration, reaction time, and extract ratio on biogenic AgNP formation have been reported in plant-, cyanobacteria-, and algae-mediated systems [31,32,33,34,35].

The color change from pale yellow to brown and the UV–visible surface plasmon resonance peak at approximately 440 nm provided strong evidence for AgNP formation. The absence of comparable color development in control solutions without algal extract or without AgNO_3_ further supports that nanoparticle formation resulted from the interaction between silver ions and biomolecules in the *U. reticulata* extract. The surface plasmon resonance of AgNPs is influenced by particle size, morphology, surface chemistry, and the surrounding medium; therefore, the observed absorbance peak is consistent with nanoscale silver particle formation [11,12].

FTIR analysis indicated that functional groups present in the algal extract contributed to AgNP synthesis and stabilization. The absorption region between 3500 and 3700 cm^−1^ suggests the involvement of hydroxyl and amine groups, while peaks near 1758.80 and 1037.52 cm^−1^ indicate carbonyl and C–O stretching vibrations, respectively. These groups may originate from algal polysaccharides, proteins, phenolic compounds, or other oxygen- and nitrogen-containing metabolites. Such biomolecules can donate electrons for Ag+ reduction and may adsorb onto nanoparticle surfaces as natural capping agents. Similar functional-group involvement has been reported in green nanoparticle synthesis using plant and algal extracts [17,30,36]. However, FTIR identifies functional groups rather than specific compounds; therefore, these results suggest biomolecular involvement but do not identify the exact metabolites responsible for reduction and capping.

SEM and EDS analyses further supported successful AgNP formation using *U. reticulata* extract. SEM showed irregular to predominantly spherical nanoparticles with visible aggregation, with an average particle size of approximately 3.1 nm estimated from about 100 particles. This small particle size may be beneficial for photocatalytic activity because smaller particles generally provide a larger surface-area-to-volume ratio and more active sites for interaction with dye molecules. EDS analysis showed characteristic silver signals at approximately 0.3 and 3.0 keV, confirming the elemental silver composition of the synthesized nanoparticles. Previous studies have also reported spherical AgNPs produced using marine macroalgae such as *Caulerpa serrulata*, *Padina gymnospora*, and *Sargassum ilicifolium*, although particle size varies depending on the algal species and synthesis conditions [19,20,22,27]. A comparison of seaweed-mediated nanoparticles and their reported photocatalytic applications is presented in Table S1. Zeta potential analysis indicated a near-neutral surface charge, suggesting limited electrostatic stabilization. Surface-associated algal biomolecules may nevertheless contribute to steric stabilization of the nanoparticles. Future DLS-based size-distribution and time-dependent stability analyses would further clarify the dispersion behavior of the synthesized AgNPs under storage and application conditions.

The synthesized AgNPs showed sunlight-assisted decolorization of methylene blue, methyl orange, aniline blue, and rose bengal. The time-dependent decrease in absorbance indicates that AgNPs promoted decolorization of structurally different dyes under natural sunlight. This activity may be related to the surface plasmon resonance behavior of AgNPs, which can enhance light absorption and support the formation of reactive species capable of attacking dye chromophores. The small particle size may also improve dye removal by increasing surface contact between AgNPs and dye molecules. Previous studies have similarly reported dye decolorization or degradation using green-synthesized AgNPs and seaweed-derived nanomaterials under light exposure [12,21,34,35,36,37,38,39]. The 60–90% decolorization observed after 24 h supports the potential of *U. reticulata*-mediated AgNPs for dye-contaminated water treatment.

The sunlight-assisted decolorization observed in this study supports the potential application of *U. reticulata*-mediated AgNPs in dye-contaminated water treatment. Although UV–visible absorbance provides a useful measure of dye decolorization, future studies using TOC or COD analysis, degradation-product profiling, toxicity assessment, catalyst reuse tests, and real textile effluents would further clarify the treatment efficiency and practical applicability of these macroalga-mediated AgNPs. Although the produced AgNPs showed substantial sunlight-assisted dye decolorization, the recyclability of the catalyst was not assessed in the present study. Future research should look into repeated-use cycles, catalyst recovery efficiency, Ag leaching, and structural stability to determine the practical usefulness of the AgNP photocatalyst.

## 5. Conclusions

In this study, silver nanoparticles were successfully synthesized using an aqueous extract of the marine green macroalga *U. reticulata*. Among the five seaweed species screened, *U. reticulata* was selected for further optimization based on visible color change and UV–visible spectral response. The optimized synthesis conditions were light exposure, 1 mM AgNO_3_, a 7:3 AgNO_3_-to-extract ratio, pH 8, incubation at 60 °C, and a reaction time of 24 h. UV–visible spectroscopy showed a characteristic surface plasmon resonance peak at approximately 440 nm, while FTIR analysis indicated the involvement of algal functional groups in AgNP formation and stabilization. SEM and EDS analyses confirmed the formation of predominantly spherical AgNPs with elemental silver composition. The synthesized AgNPs showed effective sunlight-assisted decolorization of methylene blue, methyl orange, aniline blue, and rose bengal, achieving approximately 60–90% decolorization after 24 h. These findings suggest that *U. reticulata* is a promising marine biological resource for green AgNP synthesis and that the resulting nanoparticles have potential for sustainable treatment of dye-contaminated wastewater.

## Figures and Tables

**Table 1:** Screening of selected seaweed species for silver nanoparticle synthesis under light and dark incubation conditions.

Name of the Seaweeds	Image of Seaweed	Incubation Condition	
		Light	Dark
*Ulva reticulata*	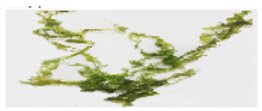	+	-
*Ulva lactuca*	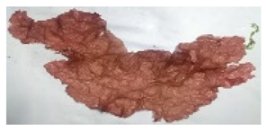	-	-
*Codium tomentosum*	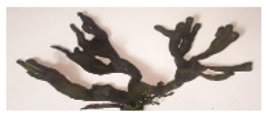	-	-
*Sargassum swartzii*	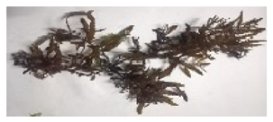	+	-
*Halymenia floresia*	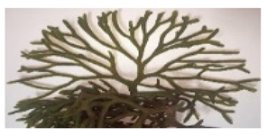	-	-

## Data Availability

The original contributions presented in this study are included in the article/Supplementary Materials. Further inquiries can be directed to the corresponding authors.

## Supplementary Materials

The following supporting information can be downloaded at https://www.mdpi.com/article/10.3390/nano16191261/s1, Table S1: Comparison of seaweed-mediated nanoparticle synthesis with photocatalytic applications. References [40,41,42,43,44] are cited in the Supplementary Materials.
