# Supplementary material for: Marine Macroalga-Mediated Green Synthesis of Silver Nanoparticles Using Ulva reticulata for Sunlight-Assisted Photocatalytic Degradation of Synthetic Dyes
Source: Nanomaterials (Basel). 2026 Oct 7;16(19):1261. doi: 10.3390/nano16191261 (PMC13667079; doi:10.3390/nano16191261)
Supplement: Supplementary file 1 [file nanomaterials-16-01261-s001.zip › nanomaterials-4493119-supplementary.pdf]

Table S1. Comparison of seaweed-mediated nanoparticle synthesis with photocatalytic applications

S. No.	Name of the seaweeds	Nanoparticles	Size (nm)	Shape (or) Morphology	Application	Reference
1	*Sargassum ilicifolium*	AgNPs	27.9	Spherical, isotropic and monodisperse.	Bactericidal and Photocatalytic activity	Devi et al., 2022
2	*Caulerpa serrulata*	AgNPs	10 ± 2	Spherical	Antibacterial and Photocatalytic activity	Aboelfetoh et al., 2017
3	*Padina gymnospora*	AgNPs	5 - 20	Spherical	Photocatalytic activity	Rajaboopathi and Thambidurai, 2018
4	*Sargassum cervicorne*	CuNPs	2.6 - 3	Spherical	Photocatalytic activity	Anwar et al., 2024
5	*Caulerpa racemosa*	AgNPs	25	Spherical	Photocatalytic activity	Edison et al., 2016
6	*Padina tetrastromatica*	ZnONPs	16.6 - 30.8	Hexagon	Photocatalytic and Antibacterial activity	Pandimurugan and Thambidurai, 2016
7	*Padina pavonica*	AgNPs	15 - 90	Spherical	In-vitro antimicrobial and Photocatalytic activity	Thankachan et al., 2024
8	*Sargassum muticum*	ZnONPs	15 - 50	Spherical	In vitro anti-microbial and Photocatalytic activity	Subramanian et al., 2022
9	*Ulva lactuca*	ZnONps	10 – 50	Asymmetrical	Photocatalytic activity, Antimicrobials and Insecticides	Ishwarya et al., 2018
10	*Ulva reticulata*	AgNPs	3.1	Spherical	Photocatalytic activity	This study

References:

1. Devi, T.A.; Sivaraman, R.M.; Thavamani, S.S.; Amaladhas, T.P.; AlSalhi, M.S.; Devanesan, S.; Kannan, M.M. Green synthesis of plasmonic nanoparticles using *Sargassum ilicifolium* and application in photocatalytic degradation of cationic dyes. *Env. Res.* **2022**, 208, 112642.
2. Aboelfetoh, E.F.; El-Shenody, R.A.; Ghobara, M.M. Eco-friendly synthesis of silver nanoparticles using green algae (*Caulerpa serrulata*): reaction optimization, catalytic and antibacterial activities. *Environ. Monit. Assess*. **2017**, 189, 1-15.
3. Rajaboopathi, S.; Thambidurai, S. Enhanced photocatalytic activity of Ag-ZnO nanoparticles synthesized by using *Padina gymnospora* seaweed extract. *J. Mol. Liq*. **2018**, 262, 148-160.
4. Anwar, S.J.; Yusoff, H.M.; Bhat, I.U.H.; Ern, L.K. Remediation of dye-contaminated water using brown algae seaweed supported copper nanoparticles. *Arab. J. Sci. Eng.* **2024**, 49(1), 475-496.
5. Edison, T.N.J.I.; Atchudan, R.; Kamal, C.; Lee, Y.R. *Caulerpa racemosa*: a marine green alga for eco-friendly synthesis of silver nanoparticles and its catalytic degradation of methylene blue. *Bioprocess biosyst. Eng*. **2016**, 39, 1401-1408.
6. Pandimurugan, R.; Thambidurai, S. Novel seaweed capped ZnO nanoparticles for effective dye photodegradation and antibacterial activity. *Adv. Powder. Tech*. **2016**, 27(4), 1062-1072.
7. Thankachan, M.K.; Ganapaty, S.; Thomas, D.; Chandrasekaran, R.; Ramachandran, S.K.; Krishnan, M. Green fused nanoparticles from seaweed as a sustainable resource: Study on antimicrobial activities against human pathogens and photocatalytic degradation of methylene blue (MB) dye. *Inorg. Chem. Commun*. **2024**, 170, 113415.
8. Subramanian, H.; Krishnan, M.; Mahalingam, A. Photocatalytic dye degradation and photoexcited anti-microbial activities of green zinc oxide nanoparticles synthesized via *Sargassum muticum* extracts. *RSC advances*. **2022**, 12(2), 985-997.
9. Ishwarya, R.; Vaseeharan, B.; Kalyani, S.; Banumathi, B.; Govindarajan, M.; Alharbi, N.S.; Benelli, G. Facile green synthesis of zinc oxide nanoparticles using *Ulva lactuca* seaweed extract and evaluation of their photocatalytic, antibiofilm and insecticidal activity. *J. Photochem. Photobiol. B-Biol.* **2018**, 178, 249-258.
